# Hybrid Materials Based on Magnetic Iron Oxides with Benzothiazole Derivatives: A Plausible Potential Spectroscopy Probe

**DOI:** 10.3390/ijms22083980

**Published:** 2021-04-12

**Authors:** Silviana Corrêa, Isael Aparecido Rosa, Gustavo A. Andolpho, Letícia Cristina de Assis, Maíra dos S. Pires, Lívia C. T. Lacerda, Francisco G. E. Nogueira, Elaine F. F. da Cunha, Eugenie Nepovimova, Kamil Kuca, Teodorico C. Ramalho

**Affiliations:** 1 Department of Chemistry, Federal University of Lavras, No 37, Lavras 37200-900, MG, Brazil; silvianacorrea123@yahoo.com.br (S.C.); isaelrosa@ufla.br (I.A.R.); gustavo.almeida.andolpho@gmail.com (G.A.A.); leticiaassisquimica@hotmail.com (L.C.d.A.); mairapires15@hotmail.com (M.d.S.P.); livia.lacerda@ifnmg.edu.br (L.C.T.L.); elaine_cunha@ufla.br (E.F.F.d.C.); 2 Department of General Formation, Federal Center of Technological Education of Minas Gerais—Varginha, Avenida dos Imigrantes, 1000, Panorama Garden, Varginha 37022-560, MG, Brazil; 3 Federal Institute of North Minas Gerais—Campus Pirapora, Avenue Humberto Mallard, 1355—Santos Dumont, Pirapora 39270-000, MG, Brazil; 4 Department of Chemical Engineering, Federal University of São Carlos, São Carlos 13565-905, SP, Brazil; nogueira@ufscar.br; 5 Department of Chemistry, Faculty of Science, University of Hradec Kralove, 50000 Hradec Kralove, Czech Republic; eugenie.nepovimova@uhk.cz

**Keywords:** hybrid material, feroxyhyte, benzothiazoles, DFT, molecular docking, spectroscopy probe

## Abstract

Rare diseases affect a small part of the population, and the most affected are children. Because of the low availability of patients for testing, the pharmaceutical industry cannot develop drugs for the diagnosis of many of these orphan diseases. In this sense, the use of benzothiazole compounds that are highly selective and can act as spectroscopy probes, especially the compound 2-(4′-aminophenyl)benzothiazole (ABT), has been highlighted. This article reports the design of potential contrast agents based on ABT and iron to develop a new material with an efficient mechanism to raise the relaxation rate, facilitating diagnosis. The ABT/δ-FeOOH hybrid material was prepared by grafting (N-(4’-aminophenyl) benzothiazole-2-bromoacetamide) on the surface of the iron oxyhydroxide particles. FTIR spectra confirmed the material formations of the hybrid material ABT/δ-FeOOH. SEM analysis checked the covering of nanoflakes’ surfaces in relation to the morphology of the samples. The theoretical calculations test a better binding mode of compound with iron oxyhydroxide. Theoretical findings show the radical capture mechanism in the stabilization of this new material. In this context, Fe^3+^ ions are an electron acceptor from the organic phase.

## 1. Introduction

There is no unique definition of what constitutes a rare disease, so each government defines the conditions that make a disease be classified as rare. In the US, the Health Promotion and Disease Prevention Amendments of 1984 define a rare disease as any condition present in less than 200,000 people. This definition is not precise worldwide; in Europe, a disease is classified as rare if it affects one person in 2000, and in Japan, the definition is one in 2500 people [1,2,3].

This fact leads to an imprecise reckoning of rare diseases. One disease can be rare in some countries or regions and non-rare in others. Several authors report that between 5000 and 8000 rare diseases are cataloged. This large number of diseases affect a small part of the population, about 5–10%—mainly children, who make up half of all patients of rare diseases. Approximately one-third of these children die before turning five years old. This feature leads to difficulty in diagnosis due the doctors’ lacking knowledge of these diseases, the low availability of orphan drugs (i.e., drugs for rare diseases), and the difficulty of conducting research about new orphan drugs [1,2,4,5].

The compounds called radiopharmaceuticals are medicines containing radionuclides, often used in nuclear medicine for the diagnosis and treatment of several diseases. A large number of radiopharmaceuticals may be employed, including complexes containing metals like magnesium [6], gadolinium [7], and others. We can also highlight compounds based on metal complexes of technetium (Tc) and its 99 mTc metastable nuclear isomer [8]. 

The derivative compounds of phenylbenzothiazole (PBT) show versatile biological behavior, especially the compound 2-(4’-aminophenyl)benzothiazole (ABT), and their chemical activity is connected to the binding mode of these molecules to the protein phosphoinositide 3-kinase (PI3K) and other kinases. By analyzing the pharmacological and physicochemical properties of these compounds, it is concluded that this class of compounds can be functionalized, forming an organic–inorganic hybrid material so that it can act as a spectroscopic probe, deliver drugs at specific sites, or provide imaging information as a sensor [6,8]. In this line, the use of iron oxide nanoparticles as the inorganic part is an alternative for biomedical applications to avoid especially high toxicity rates, due to the higher compatibility of iron with the human body [9].

Magnetic resonance is a modern and accurate non-invasive medical tool that produces detailed three-dimensional anatomical images without the application of harmful radiation. This technique uses radiofrequency (RF) radiation combined with a meticulously controlled magnetic field to generate high-quality cross-sectional images on any plane. MRI is such a powerful and accurate technique that MRI devices can provide images of internal parts of the human body so that professionals can be highly aware when evaluating patients’ examinations. It is often used for disease detection, diagnosis, and monitoring of treatment. These techniques use radiopharmaceuticals containing radionuclides emitting γ and β radiation, making it possible to obtain body images. Therefore, a hunt for new compounds with these properties is increasingly encouraged. 

After the application of radiation, the atomic nuclei of the body align themselves with the magnetic field, and then an RF signal is obtained. The energy released from the patient’s body is captured and applied to generate computer images. In order to provide a higher image contrast, contrast agents are used, which allows better observation of the tissues, providing a better exploration of their physical and structural properties [10]. 

The MRI technique is supported by a refined technology that stimulates and detects changes in the axis of rotation of protons present in the water molecules of tissues—specifically in the magnetic properties of ^1^H nuclei, because the high concentration of water in biological systems makes hydrogen atoms abundant in the human body [8]. Compounds containing paramagnetic metals can be used in therapy or diagnosis for several diseases.

However, some studies indicate that other systems containing Fe (III) and Mn (II) are promising [10]. Some of the most widely used compounds contain Gd, but although they are approved by the FDA, they can be toxic in some places, especially when applied as a contrast agent for patients with kidney, heart, and ischemic problems. Its properties grant Gd nanoparticles and iron oxide nanoparticles (IONPs) a guaranteed privileged place in biomedicine. 

IONPs have been widely used in magnetic resonance imaging (MRI) as contrast agents for diagnosis, due to their low side effects, metastasis inhibition, and prevention of resistance to drugs used in treatment [11,12]. Some published studies have shown positive results on the bioactivity of a number of drugs and antimicrobial formulations based on different metals, such as gold, silver, manganese, zinc, iron, and others, as nanoparticles [13]. 

The regulation of biological processes occurs mainly through a combination of factors: structural (including geometric structure and electronic structure), energetic, dynamic, and kinetic. Regarding the structural part, the three-dimensional aspects, which were not previously considered, now bear evidence of a substance’s behavior in the biological environment. They have mainly brought an enlarged view of the bioactive conformation of the ligand at the moment of interaction with the target protein [14]. 

With the advancement of nanotechnology, several applications in different areas of medicine have been privileged. This has provided great advances in diagnosis, biological detection, therapy, and drug delivery [15]. The application of iron oxide nanoparticles is an alternative for biomedical applications to avoid especially high toxicity rates due to the higher compatibility of iron with the human body. Allied to this is its higher efficiency, which allows a smaller dose to be applied to the patient. 

The process of developing a new drug is long, difficult, expensive, and risky. To succeed in this process, it takes about 10 years and a cost that exceeds USD 1 billion. Therefore, the methodologies that use computational tools aim to facilitate and optimize the development of new compounds, reducing costs and avoiding the waste of manpower and resources. This technique has been growing steadily in recent years [6,13].

Among iron oxides, δ-FeOOH has gained special prominence because it is stable in the biochemical environment. This oxide is a polymorph of common iron oxyhydroxides and its structure is similar to hematite (α-Fe_2_O_3_), presenting a hexagonal oxygen network with half of the available octahedral interstices filled with iron [7].

Because it has superparamagnetic properties, δ-FeOOH has great potential for use in medicine. However, few detailed works, whether computational or experimental, have been published about it. Therefore, this work aims to elucidate the morphological, structural, and other properties of ABT/δ-FeOOH using experimental and theoretical techniques with structural and electronic parameters.

## 2. Results

**Figure 1:**
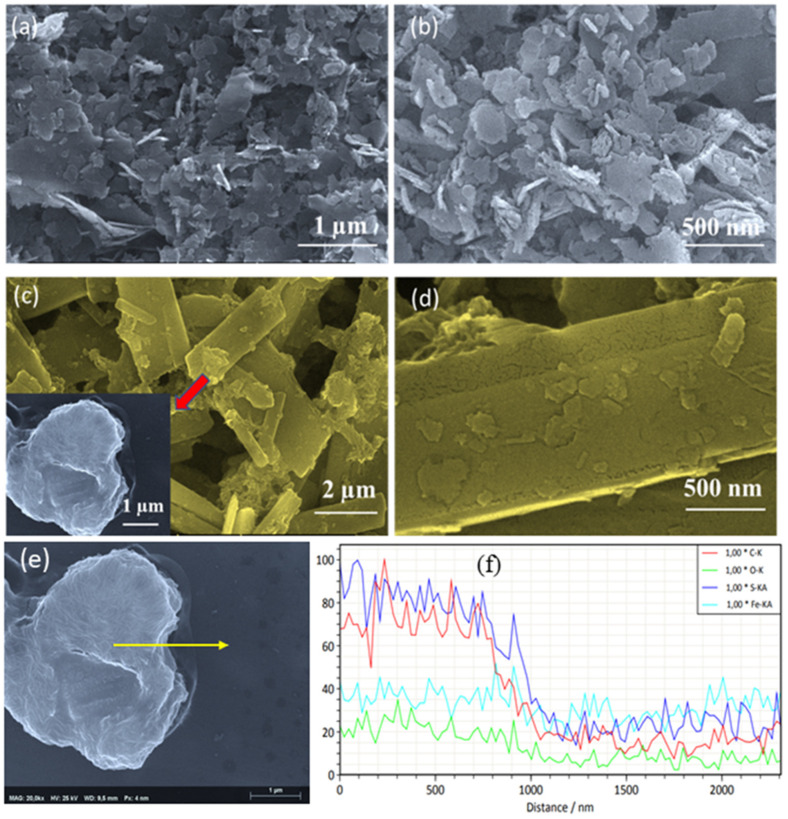
SEM images of (**a**,**b**) δ-FeOOH, (**c**,**d**) ABT/δ-FeOOH; (**e**) SEM image with a yellow arrow indicating the EDX line scan position; (**f**) the relative intensities of the different elements (i.e., C, O, S, and Fe) obtained by EDX line scan on the surface of ABT/δ-FeOOH material.

The scanning electron microscopy (SEM) images of the δ-FeOOH pure and functionalized material are shown in Figure 1.

**Figure 2:**
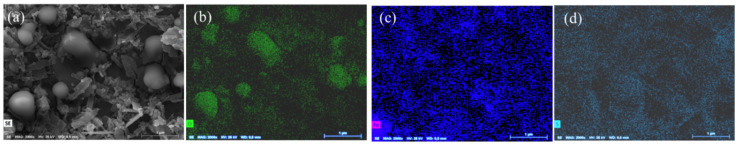
EDX mapping images of ABT/δ-FeOOH material (**a**) SEM image corresponding to (**b**) Oxygen, (**c**) Iron and (**d**) Sulfur elements.

Additionally, to analyze how the elements S and Fe are distributed in the functionalized material, EDX mapping was accomplished, as seen in Figure 2.

**Figure 3:**
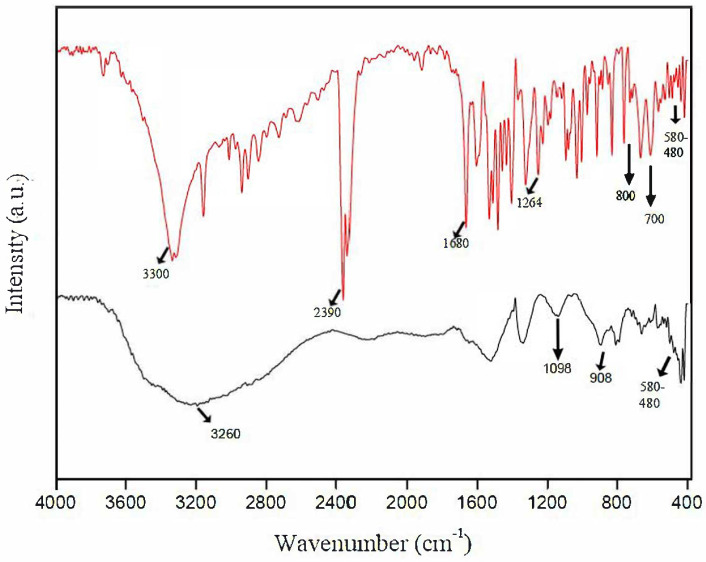
The FTIR spectra of δ-FeOOH/ABT (red line) and δ-FeOOH (black line).

The characterization of the surface groups present in the materials was performed by Fourier transform infrared spectroscopy. The result is presented in Figure 3.

**Figure 4:**
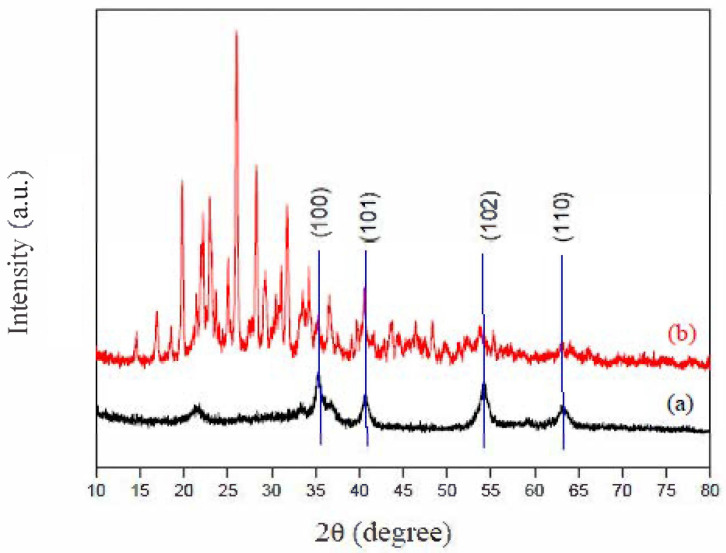
XRD patterns of (**a**) δ-FeOOH and (**b**) ABT/δ-FeOOH materials.

In order to more effectively analyze the influence of the presence of ABT molecules on the surface of the synthesized iron oxide phase, powder XRD analysis was performed. Figure 4 shows the XRD patterns of the δ-FeOOH and ABT/δ-FeOOH materials.

### Theoretical Studies

**Figure 5:**
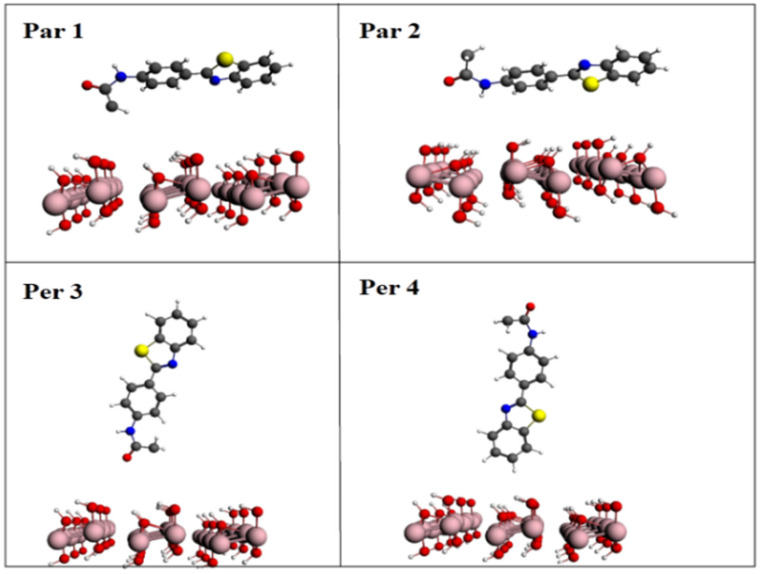
Schematic view of the derivative ABT interacting with the feroxyhyte surface in four arrangements: **Par1**: the functional group interacts in parallel with the surface; **Par2**: the functional group interacts in parallel with the surface; **Per3**: the ABT functional group interacts perpendicularly with the surface; **Per4**: the ABT functional group interacts perpendicularly with the surface.

DFT calculations were applied to examine the binding mode of the ABT derivative molecule by approaching this molecule to the surface of feroxyhyte in two ways, that is, the ABT derivative molecule parallel (Par1 and Par2) or perpendicular (Per3 and Per4) to the feroxyhyte surface (Figure 5). 

**Figure 6:**
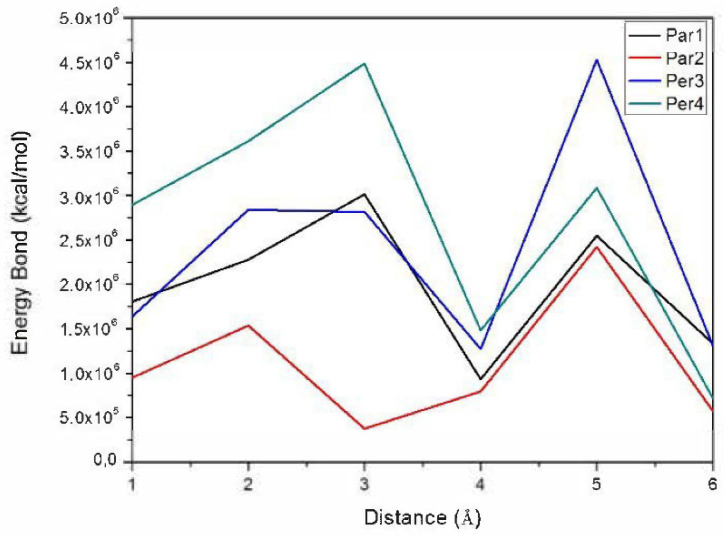
Evolution of energy values at different distances of the derivative ABT molecule and the feroxyhyte surface.

Figure 6 presents plots of the energy values calculated for the different ABT/δ-FeOOH configurations.

**Figure 7:**
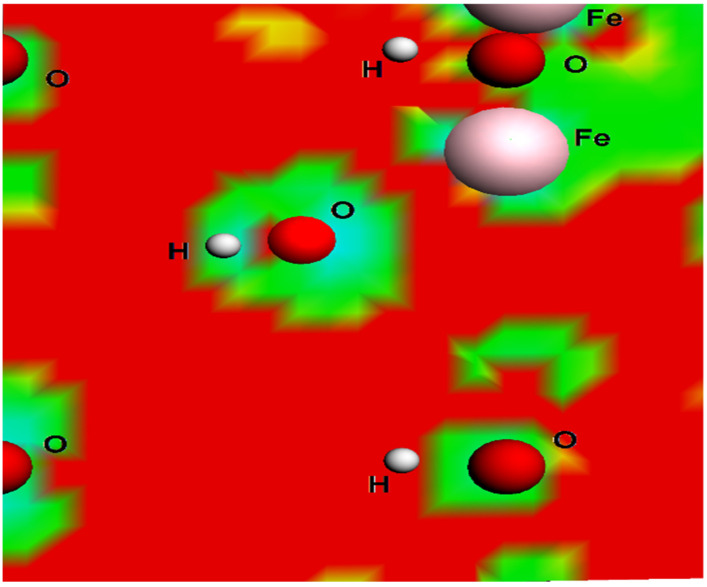
Electrostatic surface contours of δ-FeOOH plane (100). Red and green indicate volumes of low electron density and high electron density, respectively.

The calculated density state map is shown in Figure 7, which was observed in the present study for the plane (100) [16]. The low electron density is indicated by the red areas around the iron atoms. These electrostatic calculations point to Fe as the location where the ABT molecule preferentially binds. 

The three-dimensional structure of the ABT/δ-FeOOH complex was optimized, and then the atomic charges were obtained by the DFT method, B3LYP/6-31G (d,p) in the Gaussian program [17]. The crystallographic structure coordinates of the PI3K enzyme were obtained from Protein Data Bank (PDB code: 3QJZ) [18], which presents the active ligand N-{6-[2-(methylsulfanyl) pyrimidin-4-yl]-1,3-benzothiazole-2-yl} acetamide. Molecular modeling was also performed on the missing residues of the enzyme using the Swiss Model program [19]; then, hydrogen atoms were added using the Discovery Studio program and protonated on the H++ server [20]. After this preparation, the Molegro Virtual Docker (MVD) [21] program was used for the docking study, in order to investigate the intermolecular interactions between the complex and the enzyme. 

Molecular docking generates a model for the development of new ligands from the structure of the target site of the biomacromolecule, estimating the binding affinity between the ligand and its receptor. Docking simulations were applied using the optimized algorithm MOLDOCK, implemented in MVD, which is capable of accurately identifying the probable conformations and orientations of the complex (poses) at the enzyme interaction site. The binding site was restricted within a 12 Å radius sphere for fit study. In addition, multiple executions were carried out for the ABT/δ-FeOOH complex, generating 100 poses, which was necessary to avoid random results because of the stochastic nature of the nesting algorithm.

**Figure 8:**
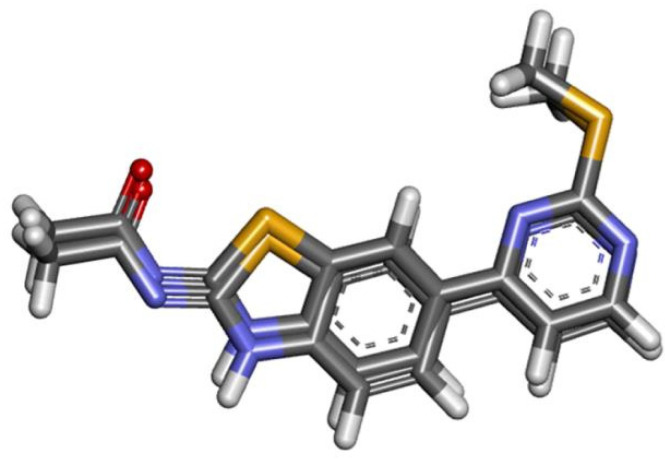
Re-docking of the active ligand at the PI3K site.

Molecular docking was performed to elucidate the ABT/δ-FeOOH complex’s interaction mode with PI3K. In the first part of this investigation, the crystallographic ligand was redocked in the PI3K enzyme in order to assess the algorithm’s ability to predict possible ligand orientations (Figure 8).

**Figure 9:**
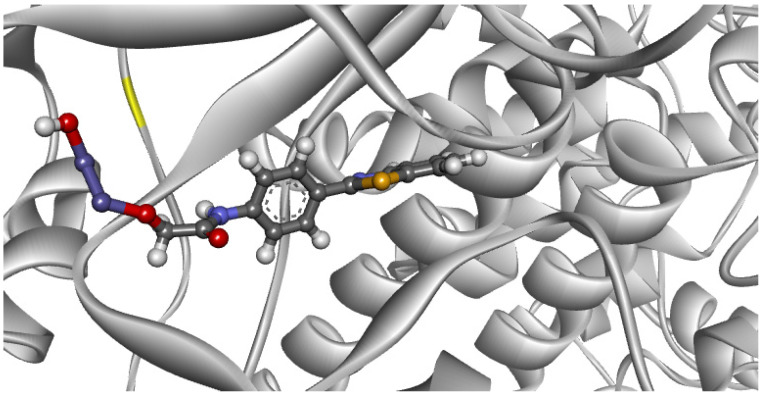
The ABT/δ-FeOOH complex within the PI3K enzyme site.

Then, we employed the best parameters provided by the data from the re-docking study to simulate the way in which our ABT/δ-FeOOH complex interacts with the PI3K enzyme, as shown in Figure 9.

**Figure 10:**
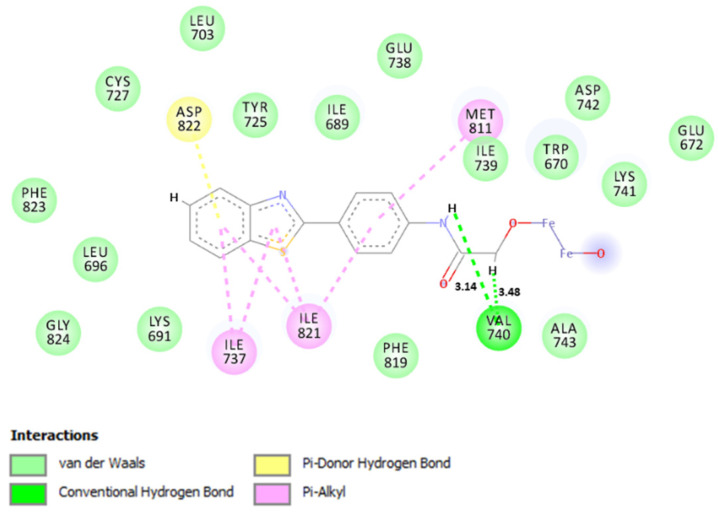
Interactions performed in molecular docking simulation with the ABT/δ-FeOOH–PI3K system.

To better analyze the molecular interactions that occurred during the docking simulation, Figure 10 was produced.

## 3. Materials and Methods 

### 3.1. Synthesis of Nanoparticles 

The δ-FeOOH particles’ synthesis was executed using a coprecipitation method following a modified procedure described by Corrêa and collaborators through the precipitation of Fe^2+^ aqueous solution using Fe(NH_4_)_2_(SO_4_)_2.6_H_2_O with NaOH (green solution) followed by the rapid oxidation of Fe^2+^ to Fe^3+^ with H_2_O_2_ (50%) [16]. This generated a brown solution after washing and drying, enabling the direct attainment of the δ-FeOOH. 

**Figure 11:**
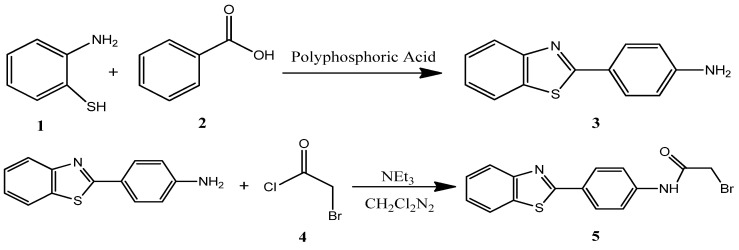
Synthesis scheme for 2-(4’-aminophenyl)benzothiazole (ABT, **3**) and compound **5**.

As displayed in Figure 11, the derivative of benzothiazole, (N-(4’-aminophenyl) benzothiazole-2-bromoacetamide), was obtained in two steps using 2-aminothiophenol as a starting material by oxidative condensation with 4-aminobenzoic acid to obtain 2-(4’-aminophenyl)benzothiazole (ABT). Compound **5** was synthesized after acetylation of ABT with bromoacetyl chloride [16].

### 3.2. Characterizations of Materials

The XRD patterns of materials were obtained by X-ray diffraction using a diffractometer (XRD 6000, Shimadzu), with Cu radiation (l = 1.54056 Å) at 2θ = 10°–80°operated at 30 mA and 40 kV. The morphology was determined by scanning electron microscopy using a Philips^®^ XL-30 FEG microscope. Fourier transform infrared spectroscopy (FTIR) was used to characterize the surface groups present on the materials. For this purpose, a PerkinElmer Spectrum 2000 Spectrometer was used to acquire the data in the 4000–400 cm^−1^ region while continuously purged of N_2_ gas to reduce the interference.

### 3.3. Computational Details 

The density functional theory (DFT) method was applied for all calculations with the software ADF-BAND 2009.01 [22]. The feroxyhyte surface and the ABT derivative molecule (Compound **5**) were studied to get a deeper understanding of how they interact. The exchange-correlation functional in the generalized gradient approximation (GGA) can be expressed using both the local electron density and the gradient of the electron density. Previous conclusions point out that the GGA functionals can provide more accurate results [22]. The optimization calculations were carried out using the PBE/GGA functional. This functional was applied with the TZP basis set, which is a large uncontracted set of Slater-type orbitals containing diffuse functions. This basis set, which is of triple-z quality, was improved with one polarization functional set: 3 d on carbon, 4 f on iron, and 2 p on hydrogen [23]. 

The δ-FeOOH structure was built using parameters based on previous studies, with space group P-3m1. It has only Fe^3+^ atoms at the octahedral sites “0, 0, and 0” and “0, 0, and 1/2.” The positions of O and H atoms are defined by their coordinates “1/3, 2/3, and 0.2468” and “1/3, 2/3, and 0.51”, and the lattice parameters a = 2.946 Å and c = 4.552 Å [16]. 

## 4. Discussion

Figure 1a shows the presence of nanoflakes with irregular form in pure δ-FeOOH. However, after functionalization with ABT, there is a complete change in the morphological features, where it is possible to observe the covering of the nanoflakes’ surface, probably due to the presence of the ABT derivative (Figure 1b). It is important to mention that the elemental composition measured across the particle (EDX line scan) for the ABT/δ-FeOOH material (Figure 1f) shows that in the ABT/δ-FeOOH surface, there is a strong enhancement to the carbon (C) and sulfur (S) signal when compared to the iron (Fe) signal due to the presence of organic compounds. 

Additionally, the elemental mapping images reveal that S and Fe elements are uniformly distributed on the δ-FeOOH functionalized material (Figure 2). It is also possible to observe an oxygen-rich region. The presence of the sulfur (S) and oxygen-rich region can be attributed to the groups of the molecule 2-(4’-aminophenyl)benzothiazole on the material surface.

Figure 3 shows the FTIR spectra of the δ-FeOOH and ABT/δ-FeOOH material samples. The FTIR spectrum for δ-FeOOH shows broadband between 3600 and 3000 cm^−1^ due to the hydroxyl groups present on its surface. On the other hand, the FTIR spectrum for ABT/δ-FeOOH exhibits well-defined bands between 3600 and 2700 cm^−1^, which can be attributed to O–H, N-H, and C–H (2930–2900 cm^−1^) stretching. The band present at 1680 cm^−1^ corresponds to the medium peak of the carbonyl stretching (C=O). It is also important to note that the bands at 1096 cm^−1^ and 908 cm^−1^ correspond to Fe–O–H bending vibrations, and those between 580 cm^−1^ and 480 cm^−1^ represent Fe–O stretch. The two benzene rings with para and ortho position substitutions show signals between 850 and 700 cm^−1^ [24].

All ABT FTIR peaks were observed in the ABT/δ-FeOOH spectrum, including the peak ν = 1264 (C=S) cm^−1^, which indicates that the ABT molecules were not fully connected to the nanoparticles of δ-FeOOH through the benzothiazole group, as suggested in the study by Benvidi [25].

Figure 4 shows the XRD patterns of the δ-FeOOH and ABT/δ-FeOOH materials. In Figure 4a, the main crystalline phase was identified as δ-FeOOH according to JCPDS File 13–87 (Figure 4a). However, after modifying the material, it is possible to observe several peaks on the surface of δ-FeOOH, probably due to the organic compounds formed during the synthesis (Figure 4b).

XRD patterns also show an agreement with the characteristic diffraction planes, (100), (101), (102), and (110), of feroxyhyte reported by Cornell et al., the first being the most intense. Theoretical calculations with the DFT method were applied to study the feroxyhyte surface before the functionalization of the ABT molecules. The results showed that the diffraction plane (100) presented lower energy, indicating higher stability [26]. 

After that, the DFT method was used to evaluate the binding modes of the ABT and δ-FeOOH molecules and the energy values for these configurations. Compared to the perpendicular configurations, the energy values for both parallel configurations, Par3 and Par4, suggested higher energy values, as shown in Figure 5 and Figure 6, respectively. The effective contact area between the ABT molecule and the feroxyhyte surface, as well as the atomic correlation between ABT and the feroxyhyte surface, can, in principle, generate some energy modification. In a previous study, Corrêa et al. pointed out that the charge density is higher when near to O atoms, which can lead to electron transfer from Fe to O atoms in iron oxides. These results are in agreement with the calculated density states map (Figure 7) [16]. 

The way in which this molecule binds to the protein PI3K is related to its chemical activity. Previous results from Mancini’s work show the interaction mode of the ABT complex at the active site. The importance of metal in the formation of hydrogen bonds with some amino acid residues (e.g., Lys800, Thr827), and the adaptation of ABT to the PI3K protein, which is present in several cellular activities, are highlighted [6,8,27].

The RMSD (Root-mean-square deviation) value for the superposition was 0.84 Å for PI3K, showing that the Molegro software is efficient in reproducing the conformation of the crystallographic ligand. The literature reports that values less than 2.00 Å are acceptable [28,29]. The re-docking overlap is presented in Figure 8.

As shown in Figure 9, the ABT/δ-FeOOH complex showed a high interaction with the PI3K active site, with interaction energy values in the range of −1976.21 kcal mol^−1^. Note that the studied compound had a higher affinity for PI3K than the co-crystallized ligand (the latter showing an interaction energy value of −64.685 kcal mol^−1^). This theoretical result of intermolecular interaction energy indicates greater stability of the ABT/δ-FeOOH complex in the active site of the enzyme when compared to the active ligand. However, it is necessary to emphasize that the results obtained do not take pharmacokinetic properties into account. Therefore, in vitro tests can contribute to evaluating immunotoxicity.

Analyzing the interactions that occurred during the docking simulation (Figure 10), it was observed that the ABT/δ-FeOOH complex performed several intermolecular interactions in the molecular docking simulation. More specifically, the ABT/δ-FeOOH complex formed two hydrogen bonds with Val740 in addition to short-range hydrophobic interactions with Met 811, Ile 821, Ile 737, and Asp 822. However, we did not observe interactions with the metal. In line with that, our results suggest that the addition of δ-FeOOH in the ABT ligand can help in accommodating the complex in the enzyme site.

Turning the inorganic phase of the hybrid material (ABT/δ-FeOOH), Gonçalves and co-workers, in 2015 [30], described the first studies of how the δ-FeOOH is able to get the relaxation time of water solvent molecules. This fact was proved by analyzing the increased value of the hyperfine coupling constant (A_iso_) of hydrogens and oxygens of water molecules in the presence of δ-FeOOH. This same group published a newer study in 2017 [7] revealing that δ-FeOOH and other different phases of iron oxides are able to increase the relaxation time of water molecules, and that they can therefore be employed as potential contrast agents for MRI.

More recently, in 2020, a new study [31] showed that the use of hybrid materials using δ-FeOOH conjugated to gadolinium contributed to a large increase of Aiso parameters when compared with the pure Gd compound. They reported that for [Gd(DTPA)(H_2_O)]^2-^, the Aiso values for ^1^H went from 0.65 MHz to 4.25 MHz, and for ^17^O, the Aiso value changed from 0.75 MHz to 5.30 MHz when it was linked to δ-FeOOH. When they studied the δ-FeOOH (100).[Gd(DTPA-BMA)(H_2_O)], the Aiso values increased by 3.30 MHz for ^1^H and 4.58 MHz for ^17^O when compared to [Gd(DTPA-BMA)(H_2_O)].

These theoretical findings put in evidence that δ-FeOOH significantly alters Aiso values, and the compound ABT interacts strongly with the protein PI3K. Therefore, the hybrid material ABT/δ-FeOOH could be a potential spectroscopy probe for rare diseases.

## 5. Conclusions

The hybrid material δ-FeOOH/ABT was successfully prepared. The molecules’ ABT particles were scattered in the iron oxide surface, as confirmed by the SEM results. The FTIR results indicated the presence of bands and peaks confirming the synthesis of the material and further suggested that the ABT molecule binds via the δ-FeOOH nanoparticle oxygens. It was clearly observed, through the surface charge density map, which regions with probability of this type of interaction were present.

Computational studies allied with experimental studies provide an excellent understanding of ABT/δ-FeOOH, both structurally and electronically.

## Data Availability

N/A.
